# Optimized Forecasting Method for Weekly Influenza Confirmed Cases

**DOI:** 10.3390/ijerph17103510

**Published:** 2020-05-18

**Authors:** Mohammed A. A. Al-qaness, Ahmed A. Ewees, Hong Fan, Mohamed Abd Elaziz

**Affiliations:** 1 State Key Laboratory for Information Engineering in Surveying, Mapping and Remote Sensing, Wuhan University, Wuhan 430079, China; alqaness@whu.edu.cn; 2 Department of e-Systems, University of Bisha, Bisha 61922, Saudi Arabia; ewees@du.edu.eg; 3 Department of Computer, Damietta University, Damietta 34517, Egypt; 4 Department of Mathematics, Faculty of Science, Zagazig University, Zagazig 44519, Egypt; meahmed@zu.edu.eg

**Keywords:** public health, forecasting, ANFIS, sine cosine algorithm, flower pollination algorithm, weekly influenza confirmed cases

## Abstract

Influenza epidemic is a serious threat to the entire world, which causes thousands of death every year and can be considered as a public health emergency that needs to be more addressed and investigated. Forecasting influenza incidences or confirmed cases is very important to do the necessary policies and plans for governments and health organizations. In this paper, we present an enhanced adaptive neuro-fuzzy inference system (ANFIS) to forecast the weekly confirmed influenza cases in China and the USA using official datasets. To overcome the limitations of the original ANFIS, we use two metaheuristics, called flower pollination algorithm (FPA) and sine cosine algorithm (SCA), to enhance the prediction of the ANFIS. The proposed FPASCA-ANFIS is evaluated using two datasets collected from the CDC and WHO websites. Furthermore, it was compared to some previous state-of-the-art approaches. Experimental results confirmed that the FPASCA-ANFIS outperformed the compared methods using variant measures, including RMSRE, MAPE, MAE, and $R^{2}$.

## 1. Introduction

Influenza is a severe threat to worldwide public health. Many people in the world get influenza annually, for example, in the USA, about 7% of the adults and 20% of children under five years of age [1]. By analyzing the historical influenza outbreaks in the USA, we can find the serious effects of influenza on the public health of the USA, as well as to the entire world. Based on the Centers for Disease Control and Prevention (CDC) data, influenza causes greater than 2% of the deaths in the USA, which can be considered the highest mortality compared to other infectious diseases. It causes about 3000 to 50,000 deaths per year in the USA, and 500,000 per year worldwide [2]. Therefore, it is necessary to build an efficient and reliable forecasting method for weekly influenza confirmed cases, which may help public health organizations and governments to make necessary preparations.

In the literature, many studies have been presented to forecast and predict some epidemics, including influenza, using various technologies. For example, Moss et al. [3] used the SEIR compartment model with a particle filter for forecasting influenza in Melbourne, Australia. In [4], the authors addressed the geographic spread of influenza in the USA. They proposed a forecasting model for the spatiotemporal spread of flu by considering the human mobility data. Senanayake et al. [5] proposed a Gaussian process (GP) regression model to predict the spatio–temporal propagation of influenza in the USA. Achrekar et al. [6] used Twitter data to predict influenza trends. They monitored and tracked tweets for two years (2009–2010) to conclude the spread and emergence of the influenza epidemic. Alkouz et al. [7] used Twitter data to predict influenza trends in the United Arab Emirates (UAE). Furthermore, Wang et al. [8] employed partial differential equation (PDE) and twitter data (tweets) to forecast regional influenza. Morita et al. [9] presented an influenza forecasting model compartmental model-inference system. They used reported data of influenza in the USA. In [10], several data sources were utilized to forecast influenza in the USA, including tweets, google searches, and participatory surveillance system. Shaman et al. [11], presented a seasonal outbreak of influenza forecasting method depended on an ensemble adjustment Kalman filter. They used influenza data from New York City. Moreover, in [12], another hybrid ensemble adjustment Kalman filter model was applied for seasonal influenza forecasting. Furthermore, Yang et al. [13] developed network models to forecast the outbreaks of influenza in the Neighborhoods of New York City. Gao et al. [14] used the auto-regressive integrated moving average (ARIMA) to compare predicted influenza-like illness (ILI) rates in the USA. Volkova et al. [15] proposed a long short term memory (LSTM) neural network approach with social media data to forecast influenza for military populations. Furthermore, Cao et al. [16] proposed dynamic linear models with multi-streams data to predict influenza epidemics in Shenzhen, China.

The Adaptive Neuro-Fuzzy Inference System (ANFIS) [17] is an efficient method used in various time-series forecasting applications. The ANFIS merges the prosperities of neural networks and fuzzy logic, therefore, it is flexible in determining non-linearity in time-series data. During past decades, it has been employed in numerous research fields, including time-series forecasting and prediction. For instance, forecasting stock prices [18], electricity prices [19], stock market prices [20], energy consumption [21], the performance of the product development [22] and return products [23].

However, the traditional ANFIS has a certain shortcoming in estimating its parameters, and this challenge opened a new research direction to enhance its performance by hybridizing it with other techniques, such as in [24], a hybrid wavelet-ANFIS model was proposed for forecasting meteorological drought. In [25], a hybrid model of ARIMA and ANFIS for forecasting energy consumption is presented. Wei et al. [18] proposed a hybrid of empirical mode decomposition and ANFIS for forecasting electricity prices. In recent years, metaheuristics (MH) algorithms have been widely applied in solving optimization problems, including time-series forecasting. Therefore, various MH methods have been used to improve the quality of the ANFIS. For instance, a hybrid of ANFIS and sine-cosine algorithm (SCA) for forecasting oil consumption [26]. The particle swarm optimization (PSO) [27] also was applied with ANFIS for forecasting biochar yield [27], and others electricity prices [28], and financial forecasting [29]. Other MH algorithms also applied to enhance the performance of the ANFIS, such as multi-verse optimizer (MVO) [30], salp swarm algorithm (SSA) [31], Social-spider optimization [32], and genetic algorithm (GA).

Another problem raised from the usage of individual MH algorithms is that in searching for solutions, an individual MH algorithm sometimes stocking at local optima. To solve this challenge, hybrid MH algorithms can be applied to enhance ANFIS performance. To this end, we propose an improved ANFIS forecasting model using two MH algorithms, namely flower pollination algorithm (FPA) and sine cosine algorithm (SCA). The FPA is a nature-inspired MH, presented by [33]. It is inspired by flow pollination process of flowering plants, and it has been applied in various optimization tasks, for instances, wireless antenna design [34], feature selection [35], solar applications [36,37], and sudoku puzzles [38]. The SCA is also an MH algorithm presented by [39], and it also has been widely utilized in solving optimization problems, such as job scheduling [40], image segmentation [41], time-series forecasting [26], and global optimization problems [42].

In this study, a hybrid MH method of FPA and SCA is employed to enhance the ANFIS performance. The SCA is used as a local search for the FPA. The proposed method, called FPASCA-ANFIS begins by receiving the historical weekly influenza data. Thereafter, it generates a set of solutions, in which each solution represents the value of ANFIS parameters, and the fitness value of each solution is calculated. Then, the probability of each agent is calculated. After that, the best solution is selected based on the agent that reaches the best fitness value. The best agent is updated using local or global strategy in the FPA. Then, based on the probability of each agent, the operators of SCA or FPA will be utilized in the case of the local strategy. Finally, the agent updating process is repeated till meeting the terminal criteria, where the best parameters will be applied for forecasting weekly influenza cases.

The main contributions of this paper are:

- We propose an optimized ANFIS model for forecasting weekly influenza confirmed cases using FPA and SCA.
- Data of two different countries, namely, China and the USA, were used to assess the proposed FPASCA-ANFIS.
- Extensive comparisons were implemented to evaluate the performance of the FPASCA-ANFIS. The comparative outcomes approved the efficiency of the proposed method.

The rest sections of this paper are arranged as: in Section 2, the principles of the ANFIS, SCA and FPA are presented. The FPASCA-ANFIS method is described in Section 3. In Section 4 and Section 5, we present the experimental results and the conclusion, respectively.

## 2. Preliminaries

### 2.1. ANFIS

**Figure 1:**
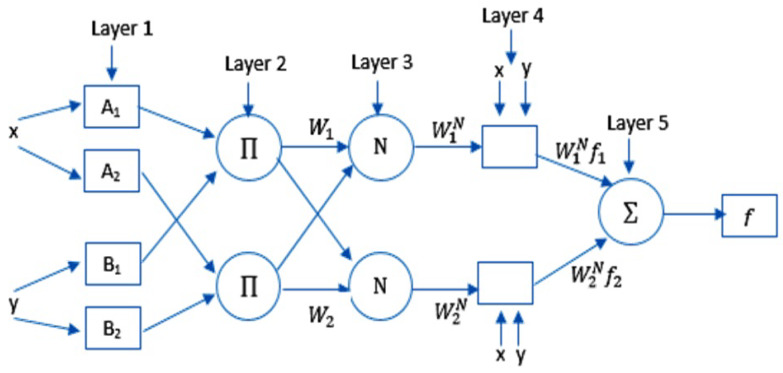
ANFIS model structure.

In this section, the preliminaries of the ANFIS are described. In general, the ANFIS combines both neural networks and fuzzy logic systems [17]. It uses a technique, called Takagi–Sugeno inference mode, or IF-THEN rules, in which a mapping between input and output is generated. Figure 1 shows the structure of the ANFIS, where *x* and *y* represent the inputs to Layer 1. $O_{1i}$ is the output of the node *i* which is represented as:

(1) $$O_{1i}=\mu_{A_{i}}\left(x\right),\;i=1,2,\;O_{1i}=\mu_{B_{i-2}}\left(y\right),\;i=3,4$$

(2) $$\mu \left(x\right)=e^{-{\left(\frac{x-\rho_{i}}{\alpha_{i}}\right)}^{2}},$$

here, the μ is the generalized Gaussian membership functions. The membership values of μ are represented by $A_{i}$ and $B_{i}$. Where $\alpha_{i}$ and $\rho_{i}$ represent the premise parameters set.

Moreover, the output of the Layer 2 (the firing strength of a rule) is computed as illustrated in Equation (3):

(3) $$O_{2i}=\mu_{A_{i}}\left(x\right)\times \mu_{B_{i-2}}\left(y\right)$$

The output of the Layer 3 (is also called the normalized firing strength) is represented as:

(4) $$O_{3i}={\bar{w}}_{i}=\frac{\omega_{i}}{\sum_{\left(i=1\right)}^{2}\omega_{i}},$$

The output of the Layer 4 (an adaptive node) is computed as:

(5) $$O_{4,i}={\bar{w}}_{i}f_{i}={\bar{w}}_{i}\left(p_{i}x+q_{i}y+r_{i}\right)$$

here, the $r_{i}$, $q_{i}$, and $p_{i}$ represent consequent parameters of *i*th node.

Furthermore, the Layer 5 has one node, and the output is represented as:

(6) $$O_{5}=\sum_{i}{\bar{w}}_{i}f_{i}$$

### 2.2. SCA

The SCA was presented by [39] as an optimization method. It begins by creating a set of random solutions. Then it uses a fitness function to assess the quality of each solution. Each solution is updated by the sine or cosine functions using a random variable $r_{1}$ as in follows:

(7) $$x_{i}^{t+1}=\left\{\begin{array}{c} x_{i}^{t}+r_{2}\times sin\left(r_{3}\right)\times \left|r_{4}x_{b}^{t}-x_{i}^{t}\right|,\;r_{1}>0.5\\ x_{i}^{t}+r_{2}\times cos\left(r_{3}\right)\times \left|r_{4}x_{b}^{t}-x_{i}^{t}\right|,\;r_{1}\leq 0.5 \end{array}\right.$$

where $x_{i}$ denotes a solution *i* and $x_{b}$ denotes the best one. $r_{2},r_{3},r_{4}$ denotes random numbers at [0,1]. The $r_{2}$ works to balance between exploration and exploitation phases.

(8) $$r_{2}=a-t\frac{a}{t_{max}}$$

in which *a*, $t_{max}$, and *t* indicate the constant value, max iterations number, and current iteration, respectively.

### 2.3. FPA

The FPA is a nature-inspired method introduced by [33]. It applies two kinds of pollination, namely local pollination and global pollination. In global pollination, the pollens needs to transfer to different plants; it can be formed as follows:

(9) $$x_{i}^{t+1}=x_{i}^{t}+L\left(x_{i}^{t}-F*\right)$$

where $x_{i}^{t}$ denotes the pollen *i* at loop *t*. *L* indicates the size of step. F* denotes the best solution. Besides, Levy fly distribution is used to control the flying of insects between different plants, as in the following equation.

(10) $$L∼\frac{\lambda \Gamma (\lambda )sin(\pi \lambda /2))}{\pi }\frac{1}{s^{1}+\lambda },\left(s\gg s_{0}>0\right),\;\lambda =1.5$$

where Γ(λ) indicates the gamma function. The Levy fly is possible for long steps s>0.

The local pollination is formed as in the following equation:

(11) $$x_{i}^{t+1}=x_{i}^{t}+\epsilon \left(x_{j}^{t}-x_{k}^{t}\right)$$

where $x_{i}^{t}$ and $x_{i}^{k}$ denote pollens from a separate flower in the plant. ϵ is in [0,1].

The pollination process can be applied by the local pollination or global pollination; thence, to switch between them, the random *p* is applied.

## 3. The Proposed FPASCA-ANFIS

The proposed FPASCA-ANFIS method is described in this section. It is a forecasting method for influenza cases in China and the USA. The FPASCA-ANFIS applies the enhanced FPA to optimize ANFIS parameters.

The structure of the FPASCA-ANFIS model is based on the ANFIS layers. In this context, the input data is fed to Layer 1, whereas, Layer 5 outputs the forecasted results. The FPASCA selects the fittest weights between Layer 5 and Layer 4 in the learning stage.

The input data are formed by FPASCA-ANFIS to be in a time series form. Autocorrelation function (ACF) was used to find patterns in the input data; in this paper, 8-lags were applied. Moreover, all data are split into 75% for training, and the testing set represented by 25% as well as the clusters numbers for ANFIS are set by the fuzzy c-mean.

In the training stage, the mean square error (MSE) is considered (as in Equation (12)) to check the quality of the ANFIS parameters. It measures the error between the real and output data.

(12) $$MSE=\frac{1}{N_{s}}\sum_{i=1}^{N_{s}}{\left(T_{i}-P_{i}\right)}^{2}$$

*T* denotes the real data, and *P* denotes the output data. More so, $N_{s}$ denotes the size of the sample.

**Figure 2:**
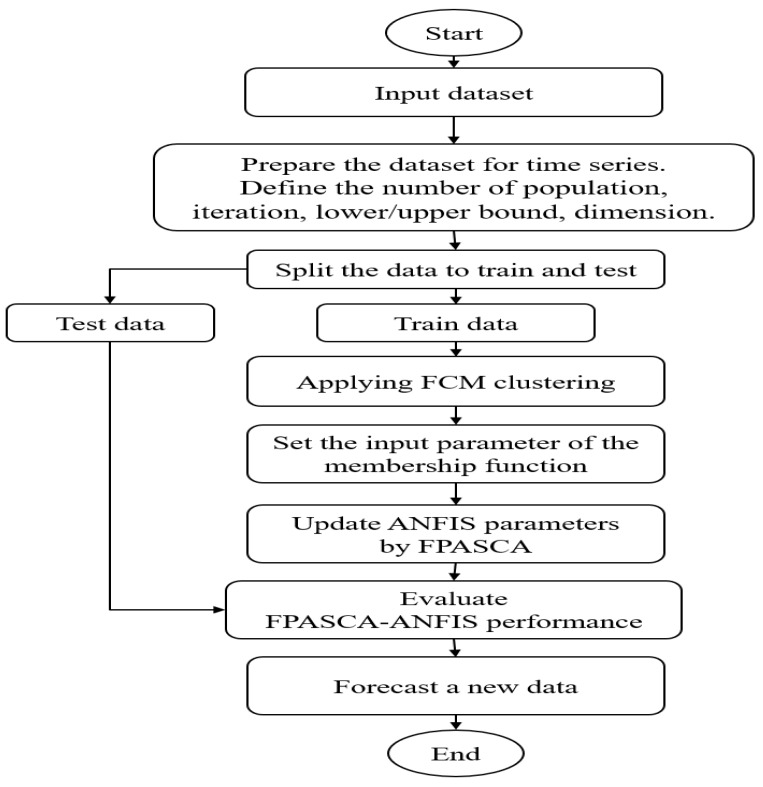
The workflow of the FPASCA-ANFIS algorithm.

The operators of the SCA are used to enhance the global pollination stage of FPA. Generally, the FPASCA-ANFIS begins by generating the problem population (*X*) then, it recalls the fitness function to evaluate each individual solution. After that, the best solution is chosen depending on the lowest MSE error and is saved to the next loop. These steps are iterated till the stop condition is met. Then, the ANFIS is trained using the best solution. Finally, the trained model is applied in the testing stage to calculate the final results. Some of the well-known measures are employed to test the performance of the FPASCA-ANFIS method. The entire stages of FPASCA-ANFIS are depicted in Figure 2.

## 4. Experimental Evaluation

### 4.1. Dataset Description

The proposed method is evaluated over two weekly influenza case datasets. The first dataset was obtained from the CDC [43]. It contains the confirmed case numbers in the USA from 2015 to 2020. The second dataset was obtained from the WHO [44]. It includes China weekly data of influenza from 2016 to 2020.

### 4.2. Performance Measures

In this subsection, five measures are employed to evaluate the performance of the FPASCA-ANFIS method, these measures are listed as follows:

- Mean Absolute Error (MAE):
  (13) $$MAE=\frac{1}{N_{s}}\sum_{i=1}^{N_{s}}\left|Py_{i}-Y_{i}\right|$$
- Mean Absolute Percentage Error (MAPE):
  (14) $$MAPE=\frac{1}{N_{s}}\sum_{i=1}^{N_{s}}\left|\frac{Py_{i}-Y_{i}}{YP_{i}}\right|$$
  Py represent the predicted value, and *Y* represent the real value.
- Root Mean Squared Relative Error (RMSRE):
  (15) $$RMSRE=\sqrt{\frac{1}{N_{s}}\sum_{i=1}^{N_{s}}{\left(\frac{Py_{i}-Y_{i}}{Py_{i}}\right)}^{2}}$$
  wherer, $N_{s}$ denotes the data size.
- Root Mean Square Error (RMSE):
  (16) $$RMSE=\sqrt{\frac{1}{N_{s}}\sum_{i=1}^{N_{s}}{\left(Py_{i}-Y_{i}\right)}^{2}}$$
- Coefficient of Determination ($R^{2}$):
  (17) $$R^{2}=1-\frac{\sum_{i=1}^{n}{\left(Y_{i}-Py_{i}\right)}^{2}}{\sum_{i=1}^{n}{\left(Y_{i}-{\bar{Y}}_{i}\right)}^{2}}$$
  in which $\bar{Y}$ denotes the mean of *Y*.

### 4.3. Results and Discussion

This subsection shows the results of the FPASCA-ANFIS in forecasting weekly confirmed cases of influenza in China and the USA. It was compared with eight existing methods, including the autoregressive integrated moving average (ARIMA), SARIMA, long short-term memory (LSTM), standard ANFIS model and the improved ANFIS using artificial bee colony (ABC), GA, FPA, and PSO. The parameters setting are set as follows, the population size is 30, and 100 loops are used. In addition, each method is evaluated for 30 runs, and the average of the measure values are recorded. The methods parameters are set as in some previous papers such as [26,30,45,46].

Table 1 and Table 2 show the results based on the testing set. Table 1 records the results of all methods in forecasting the USA influenza confirmed cases. From this table, we can see that the results of the FPASCA-ANFIS outperform the compared algorithms in almost performance metrics, except the optimization time, in which FPA is in the first rank, followed by FPASCA, and also the LSTM showed better performance in terms of MAPE and RMSRE. Meanwhile, the FPA-ANFIS and GA-ANFIS are in the second and third rank, respectively, as well as, the traditional ANFIS is ranked last. Additionally, for the optimization CPU time, the FPA-ANFIS is considered the fastest algorithm among all methods followed by FPASCA-ANFIS and PSO-ANFIS, respectively.

Table 2 presents the results of forecasted influenza confirmed cases in China. From this table, we can see that the FPASCA-ANFIS obtains the best results in all performance measures, while PSO-ANFIS, GA-ANFIS, and FPA-ANFIS, allocate the second, third, and fourth rank, respectively. Whereas, the ABC-ANFIS and ANFIS obtain the last rank. However, in terms of MAE, both FPASCA and GA have the same results. More so, in terms of MAPE and RMSRE, LSTM has the best results. For the optimization CPU time, the FPASCA-ANFIS is considered the fastest algorithm among all methods followed by FPA-ANFIS and PSO-ANFIS, respectively, whereas, the ABC-ANFIS is the slowest algorithm among the compared methods.

**Figure 3:**
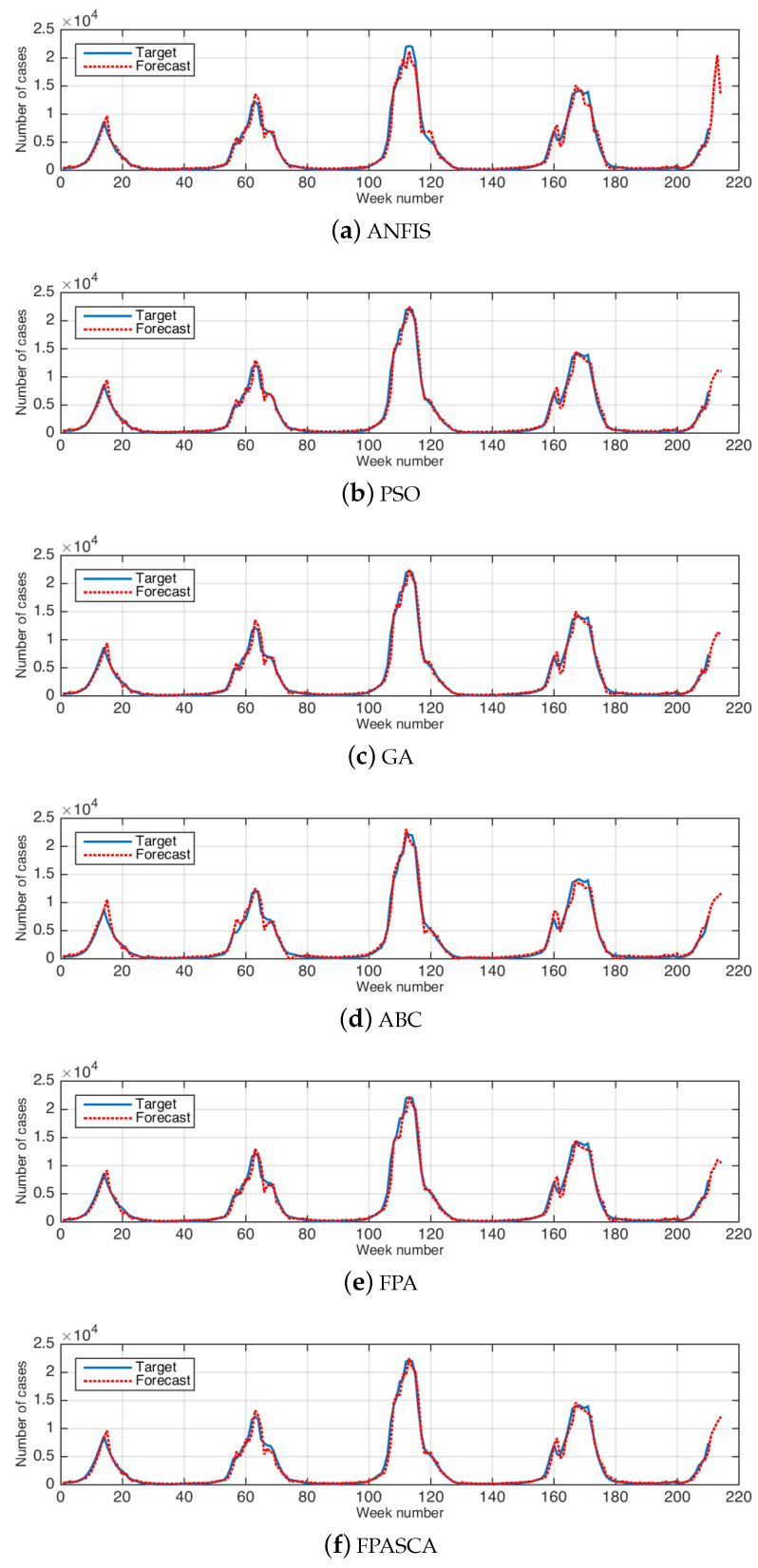
The results of real and forecasted data for influenza cases in the USA. (**a**) ANFIS, (**b**) PSO, (**c**) GA, (**d**) ABC, (**e**) FPA, (**f**) FPASCA.

**Figure 4:**
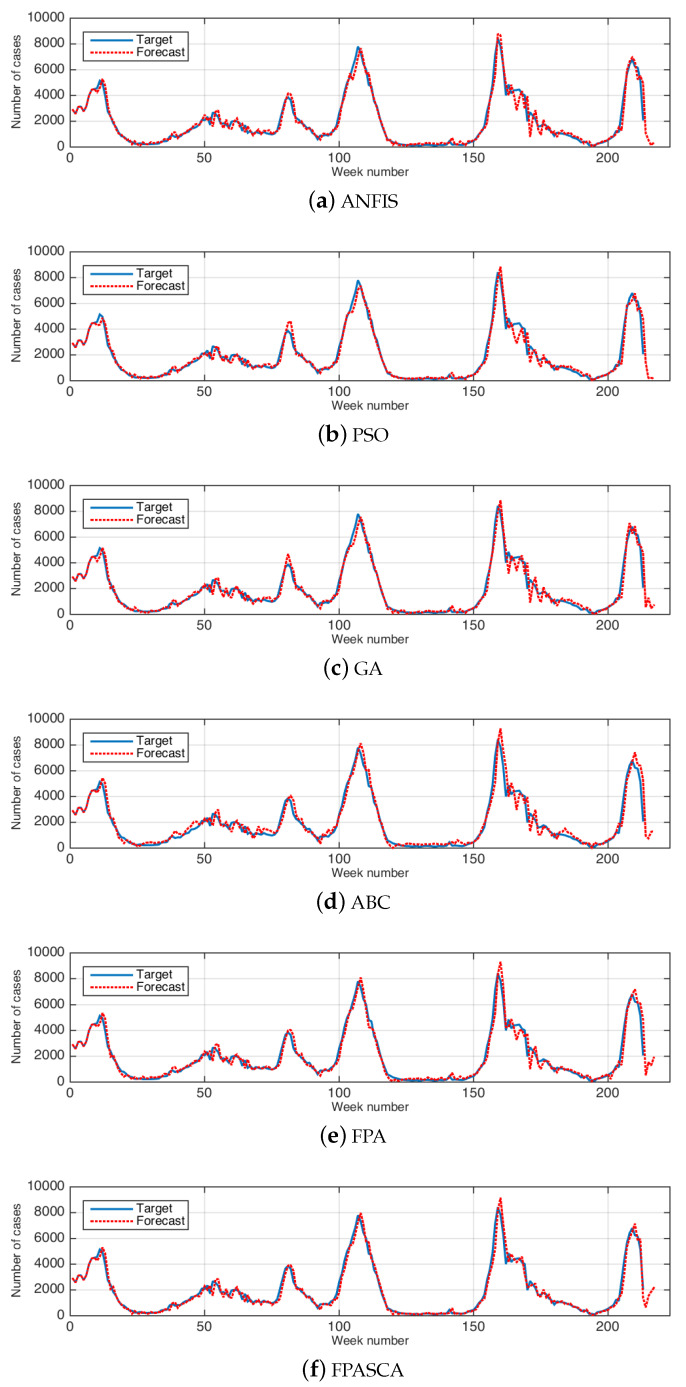
The results of real and forecasted data for influenza cases in China. (**a**) ANFIS, (**b**) PSO, (**c**) GA, (**d**) ABC, (**e**) FPA, (**f**) FPASCA.

Figure 3 and Figure 4 illustrate examples of the relation between the real data and the forecasted results in the USA and China, respectively. These figures show that the output results are closed to the real data in all weeks.

For further analysis, Wilcoxon’s rank sum test is used to test the significant difference between FPASCA and the compared methods. The results are recorded in Table 3. We can see that, in the USA dataset, there are significant differences between FPASCA and ANFIS, LSTM, SARIMA, ARIMA, PSO, GA, and ABC at level 0.05 whereas, there is no significant difference with original FPA. In China dataset, there are significant differences between FPASCA and ANFIS, GA, and ABC at level 0.05 whereas, there are no significant differences with PSO, LSTM and original FPA.

These results indicate that the FPASCA-ANFIS can effectively train the original ANFIS model and achieved better results than the compared methods.

Although the results showed that the proposed FPASCA-ANFIS has the ability to forecast the number on influenza cases based on the time-series data obtained from public datasets, in future work, there are some issues that can be addressed for further investigation and also for improving influenza cases forecasting problem. For example, the Spatio-temporal (ST) data with a self-exciting model [47,48], and also a multivariate Hawkes process (MHP) [49].

## 5. Conclusions

Governments, health organizations, and authorities strive to early detect influenza prevalence to minimize its impacts on public health. Therefore, forecasting influenza confirmed or reported cases, is an essential process that helps authorities to make the necessary plans and preparations. This study proposed an enhanced adaptive neuro-fuzzy inference system (ANFIS) using a hybrid metaheuristics (MH) method for forecasting weekly influenza cases reported in China and the USA. The hybrid MH algorithm combines both SCA and FPA, where the SCA is employed as a local search for the FPA. The hybrid FPASCA is applied to determine the optimal value for ANFIS parameters, which results in improving the ANFIS performance. The proposed forecasting method, FPASCA-ANFIS, showed high reliability in forecasting weekly cases of influenza in both mentioned countries. Moreover, comparison results showed that FPASCA-ANFIS outperforms several existing methods in terms of MAE, RMSRE, MAPE, RMSE, and $R^{2}$. Furthermore, a statistical test, the Wilcoxon test, was applied to approve the quality of the proposed FPASCA-ANFIS.

Considering the good performance of the FPASCA, it may be improved and applied in other optimization tasks, such as job scheduling, cloud computing, image processing, and feature selection.

## Figures and Tables

**Table 1:** Computational results of USA.

Method	MAE	RMSE	MAPE	R2	RMSRE	Time
ARIMA	872	1689	60.77	0.888	1.080	-
SARIMA	877	1740	54.79	0.870	0.779	-
LSTM	436	816	**27.78**	0.972	**0.354**	-
ANFIS	570	952	37.61	0.969	0.551	-
PSO-ANFIS	494	798	34.13	0.978	0.510	25.43
GA-ANFIS	480	766	35.44	0.98	0.53	28.74
ABC-ANFIS	564	878	39.79	0.972	0.593	49.27
FPA-ANFIS	411	618	37.69	0.979	0.570	**24.58**
FPASCA-ANFIS	**405**	**611**	33.64	**0.981**	0.501	25.01

the best results are in bold.

**Table 2:** Computational results of China.

Method	MAE	RMSE	MAPE	R2	RMSRE	Time
ARIMA	606	962	57.02	0.914	0.899	-
SARIMA	620	981	40.43	0.748	0.794	-
LSTM	398	623	**21.31**	0.901	**0.490**	-
ANFIS	405	718	64.21	0.858	1.198	-
PSO-ANFIS	353	620	52.07	0.892	0.871	31.64
GA-ANFIS	**362**	622	87.91	0.902	3.216	34.83
ABC-ANFIS	433	696	53.3	0.887	1.101	60.87
FPA-ANFIS	371	622	80.55	0.898	3.152	30.42
FPASCA-ANFIS	**362**	**606**	39.58	**0.906**	0.743	**25.58**

the best results are in bold.

**Table 3:** Statistical results for the FPASCA and the compared methods.

Dataset	ANFIS	PSO	GA	ABC	FPA	ARIMA	SARIMA	LSTM
USA	0.000	0.000	0.007	0.000	0.152	0.000	0.000	0.029
China	0.000	0.105	0.001	0.016	0.124	0.000	0.000	0.145
